# 3D-printed phantoms to quantify accuracy and variability of goniometric and volumetric assessment of Peyronie’s disease deformities

**DOI:** 10.1038/s41443-021-00486-9

**Published:** 2021-11-06

**Authors:** Dyvon T. Walker, Tommy Jiang, Alvaro Santamaria, Vadim Osadchiy, Doug Daniels, Renea M. Sturm, Jesse N. Mills, Sriram V. Eleswarapu

**Affiliations:** Department of Urology, David Geffen School of Medicine at UCLA, Los Angeles, CA USA

**Keywords:** Translational research, Sexual dysfunction

## Abstract

Characterization of Peyronie’s disease (PD) involves manual goniometry and penile length measurement. These techniques neglect volume loss or hourglass deformities. Inter-provider variability complicates accuracy. Using 3D-printed models, we aimed to evaluate measurement accuracy and variability and establish computational assessment workflows. Five digital phantoms were created: 13.0 cm cylinder, 13.0 cm hourglass cylinder, 15.0 cm cylinder with 40° angulation, 12.0 cm straight penis, and 12.9 cm PD penis with 68° angulation and hourglass. Lengths, volumes, and angles were determined computationally. Each phantom was 3D-printed. Ten urology providers determined lengths, angles, and volumes with measuring tape, goniometer, and volume calculator. Provider versus computational measurements were compared to determine accuracy using t-tests or Wilcoxon rank-sum tests. No significant differences were observed between manual assessment of length of penile models and designed length in penile models. Average curvature angles from providers for bent cylinder and PD phantoms were 38.3° ± 3.9° (*p* = 0.25) and 57.5° ± 7.2° (*p* = 0.006), respectively. When assessing for volume, hourglass cylinder and bent cylinder showed significant differences between designed volume and provider averages. All assessments of length, angle, and volume showed significant provider variability. Our results suggest manual measurements suffer from inaccuracy and variability. Computational workflows are useful for improved accuracy and volume assessment.

## Introduction

Peyronie’s disease (PD) is an acquired fibrotic disease characterized by plaque formation within the penile tunica albuginea. Though penile curvature is the most reported and discussed manifestation of PD, other sequelae can occur such as indentations, hourglass deformities, loss of size, and erectile dysfunction [1, 2].

The current state of diagnostics of PD involves manual goniometry to assess curvature angle and duplex ultrasonography to assess plaque characteristics. However, there is currently no standardized system for the quantification of complex PD deformities such as volume-loss deformities. Additionally, limitations to goniometry include inconsistent accuracy and precision among different providers based on provider technique [3]. Several recent groups have worked to develop novel methods for improving accuracy and precision [4–6]. For example, Margolin et al. evaluated provider variability in image acquisition of 3D printed penis models using an infrared light scanner [7]. Using five clinically relevant 3D penile models with complex deformities, we sought to evaluate inter-provider goniometer measurement variability and propose a novel 3D imaging method to reflect these metrics more accurately. We hypothesized that manual measurements would be prone to high inter-provider variability and that digital image acquisition and analysis may provide an alternative workflow for objective assessment of penile deformities.

## Methods

### Creation of digital phantoms and 3D-printed models

**Fig. 1:**
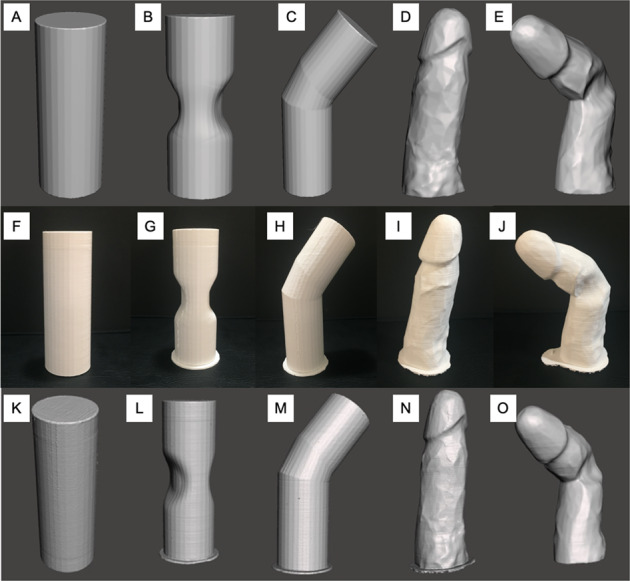
Images of original phantom creations, 3D printed models, and lightscans. **A**–**E** show original digital phantom creations. **F**–**J** show 3D-printed models. Panels **K**–**O** show 3D-printed models re-digitized by 3D structured light scanner.

Three-dimensional phantoms were created using Meshmixer (Autodesk, California, USA). These phantoms (Fig. 1, A–E) included three cylindrical models (normal cylinder, hourglass cylinder, bent cylinder) and two penile models (normal erect penis, PD model with curvature, and hourglass deformity). These digital phantoms were then 3D-printed using a Makerbot Replicator+ 3D printer (Makerbot Industries, New York, USA) using a polylactic acid polymer resin (Fig. 1, F–J). The printer had an XY positioning precision of 11 microns and a Z positioning precision of 2.5 microns. 3D models were re-digitized to evaluate the printing accuracy of the 3D printer.

### Measurements of digital phantoms

The digital phantom images were analyzed using Fusion 360 (Autodesk, California, USA). Length and volume were calculated for each phantom. Angle of curvature was measured for the bent cylinder and the PD model. The initial digital measurements served as the standard to which manual measurements would be compared. These measurement standards are shown in Table 1.

**Table 1:** Standardized measurements of original digital phantoms.

	Length (mm)	Volume (cc)	Curvature
Straight cylinder	130	203	–
Hourglass cylinder	130	187.6	–
Bent cylinder	150.4	231.9	40°
Normal penis	119.9	89.45	–
PD penis	129.3	91.97	68.1°

### Manual measurements

Manual measurements were performed on all five 3D-printed models by ten urology providers working in an andrology clinic. Length measurements were conducted using a tape measure. Measurements of angulation for the bent cylinder and the PD model were performed using a standard plastic goniometer. Providers were instructed to measure through the central plane of the object. Volumes for each model were estimated using a standardized pre-set calculator for volume of a cylinder (π*r^2^*h, where *r* is radius and *h* is height) and was calculated by each individual provider. Each provider only measured each metric once.

### 3D structured light scanning

Three-dimensional image capture was used to re-digitize each printed model (Fig. 1, K–O) using an Artec Space Spider structured light scanner (Artec 3D, Luxembourg). The Artec Space Spider utilizes blue light technology at a scanning speed of 7.5 frames per second with 0.05 mm point accuracy and 0.1 mm resolution. Images were obtained by moving the scanner around the models from a radius of ~0.3 m and were uploaded to a Dell XPS 15 laptop computer with 9th generation Intel Core i7 processor (Dell, Texas, USA), where they would be subsequently analyzed. This model of structured light scanning was chosen based on prior work that demonstrated accuracy, reliability, and precision in 3D printed blocks [6]. Measurements of length, volume, and angle were performed using Fusion 360 in similar fashion to measurements performed for the original digital phantoms.

### Statistical analysis

Means and standard deviations were calculated for all measurements. Statistical analyses were performed using R (R Foundation, Vienna, Austria). Statistical significance was defined by *p* < 0.05. Manual provider measurements of the 3D-printed models and computational measurements of the light-scanned 3D-printed models were compared to the standard digital phantom measurements using Student’s t-test or Wilcoxon rank sum test, depending on distribution. Inter-test and inter-rater reliabilities were analyzed using intraclass correlation coefficients.

## Results

**Fig. 2:**
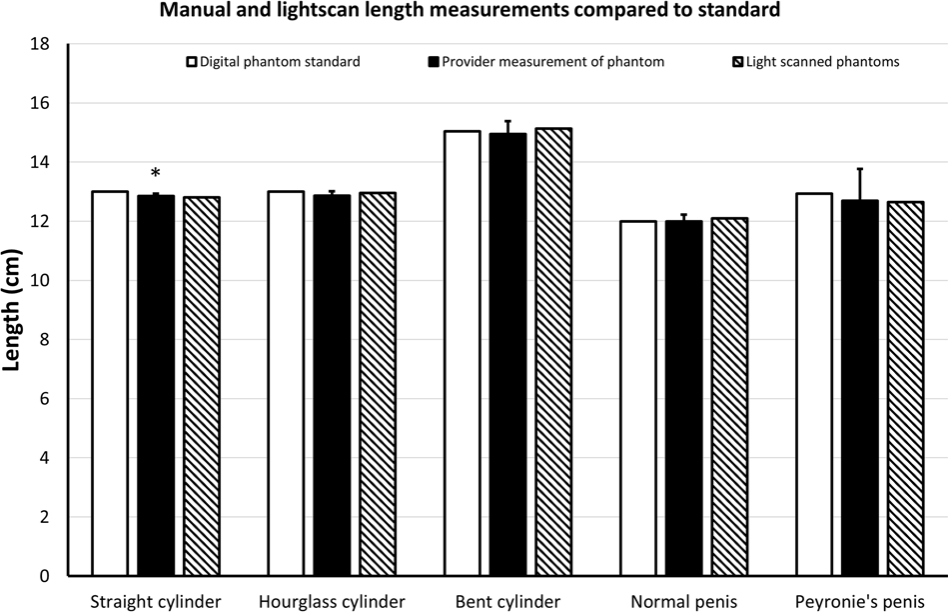
Manual and light scan length measurements compared to standard. Manual length measurements (solid black bar) and lengths of light-scanned models (hashed bar) compared to initial standard measurements of the digital phantoms (solid white bar). Wilcoxon rank-sum tests were used to compare manual length measurements or light-scanned model length measurements to the standard digital phantom measurement for each structure (* = *p* < 0.05).

**Fig. 3:**
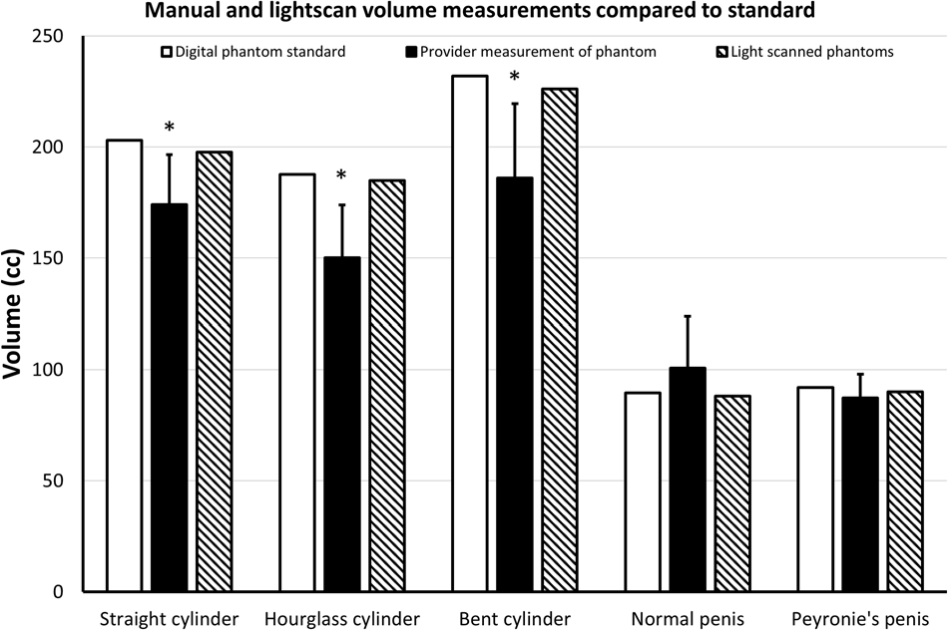
Manual and light scan volume measurements compared to standard. Manual volume measurements (solid black bar) and volumes of light-scanned models (hashed bar) compared to initial standard measurements of the digital phantoms (solid white bar) by one sample *t* test (* = *p* < 0.05).

**Fig. 4:**
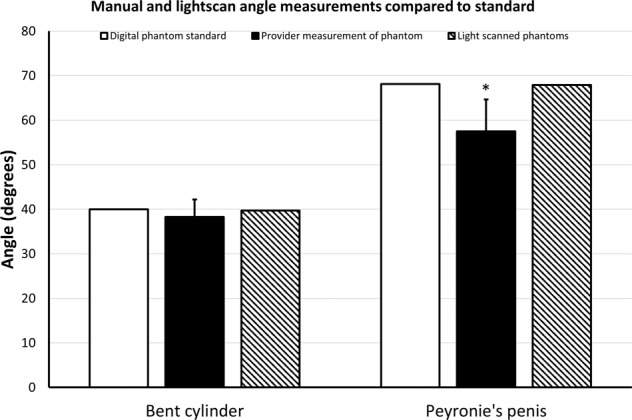
Manual and light scan angle measurements compared to standard. Manual angle measurements (solid black bar) and angle of light-scanned models (hashed bar) compared to initial standard measurements of the digital phantoms (solid white bar) by one sample *t* test (* = *p* < 0.05).

The manual length measurements for cylinder, hourglass cylinder, angled cylinder, straight penis, and PD penis were 12.9 ± 0.9 cm, 12.9 ± 1.61 cm, 15.0 ± 4.3 cm, 12.0 ± 2.3, and 12.7 ± 10.8, respectively. Comparison of these manual length measurements to the digital phantom standards revealed *p* values of *p* = 0.0003, *p* = 0.058, *p* = 0.52, *p* = 0.68, and *p* = 0.36, respectively. Figure 2 depicts these manual length measurements and the light-scanned 3D-printed model length measurements, compared to the digital phantom standards. Figure 3 depicts the manual volume measurements and the light-scanned 3D-printed model volume measurements compared to the digital phantom standards. Manual mean volumes were 174 ± 22 cc (*p* = 0.003), 150 ± 24 cc (*p* = 0.0008), 186 ± 33 cc (*p* = 0.004), 101 ± 23 cc (*p* = 0.16), and 87 ± 11 cc (*p* = 0.23), respectively. Figure 4 depicts the manual goniometric measurements and the light-scanned 3D-printed model angle measurements compared to the digital phantom standards. Manual angle measurements for bent cylinder and PD phantoms were 38.3° ± 3.9° (*p* = 0.25) and 57.5° ± 7.2° (*p* = 0.006), respectively. The discrepancy between goniometry and computationally determined angle ranged from 3° to 13°.

Table 2 shows the inter-rater reliabilities among the manual measurements of the five 3D-printed models as well as the inter-test reliabilities. The intraclass correlation coefficients were all greater than 0.75, indicating excellent reliability [8].

**Table 2:** Inter-rater and inter-test reliabilities of manual digital measurements.

Measurement parameter	Inter-rater ICC (*p*)	Inter-test ICC (*p*)
Length	0.808 (<0.01)	0.992 (<0.01)
Volume	0.769 (<0.01)	0.924 (<0.01)
Angle	0.845 (<0.01)	0.943 (<0.01)

*ICC* intra class correlation coefficient

## Discussion

Precision in measuring penile deformities in PD plays a role in determining treatment and assessing treatment efficacy [9]. Key measurements to address deformities have included penile length and angle of curvature using a goniometer. Volumetric assessment has been an underutilized metric despite patients reporting volume loss or hourglass deformities associated with PD. Inter-provider variability is proposed to be an obstacle to accurately and precisely evaluating these conditions [10]. To address inter-provider variability, we obtained measurements for length, angle, and volume of five 3D cylindrical and erect penile models to reflect clinically relevant deformities and compared this to the use of a 3D light scanner.

Penile length is typically determined via an intracavernosal erectogenic injection followed by measurement taken from the base of the penis to the tip of the penile glans using a ruler or tape measure [11, 12]. We found that providers’ measurements of length were nearly identical to each other across all the 3D-printed models. Furthermore, both the providers and 3D structured light scanner showed remarkable accuracy based on the original, standardized lengths established during creation of the digital phantoms (Fig. 2). However, given that we used static, 3D-printed polymer models, inter-provider variability in this study is likely not entirely representative of “real world” penile measurements, which are subject to changes in ambient temperature, patient comfort, pelvic musculature hypertonicity, variation in erectile rigidity, and other dynamic variables. Prior studies have attempted to measure penile length in a clinical setting to also address intra-provider variability. Sengenzer et al. conducted a study to accurately measure penile length and found stretched and flaccid lengths were able to predict erect penile length with only 65% accuracy [13]. Furthermore, Habous et al. found that inter-observer variability could account for 15–27% variability in the erect state in a study of 200 men [14]. Previous attempts to study variability and predictors of penile length have used healthy patients who were not experiencing penile deformity.

We found significant inter-provider variability and inaccuracy in angle measurement of the PD and curved cylinder 3D models (Fig. 4). Degree of curvature has been the best-studied diagnostic criterion for PD because patient satisfaction and severity of PD have often been linked to this metric [15]. Debate over the magnitude of variability between providers has long been hypothesized for penile deformities; for example, Ziegelmann et al. suggested that up to 20 degrees of variance can be elicited solely based on the provider’s subjective determination of the point of maximal curvature [3]. Indeed, contemporary non-surgical interventions for PD, such as intralesional collagenase injections or penile traction therapy, are quoted to improve penile angulation—these improvements are within the 20 degrees of variance suggested by Ziegelmann et al., and therefore the question of true efficacy becomes much more salient. Additionally, patient satisfaction has been a crucial measure for successful correction of PD, but also a measure that is highly discordant between expectation and reality [16–18]. Here we are the first study to show inter-provider variability in penile deformities using standardized, 3D-printed models that aligns closely with the hypothesis from Ziegelmann et al. These data suggest a more accurate and precise method is needed to address a crucial measurement of this condition.

PD, in many cases, also presents with volumetric changes. We found that volumetric measurement of the penis was the most susceptible to inter-provider variability for accuracy and precision (Fig. 3). For context, Margolin et al. conducted a volumetric study on 83 patients and found that 65% of PD patients experienced some level of volumetric loss which correlated to higher axial instability, psychological distress, and decreased sexual activity compared to changes in angle of curvature [19]. Additionally, volumetric and physical appearance changes in the penis were also contributory to psychiatric conditions in a self-reported analysis in PD patients [20]. The importance of volume in penile deformities is being established, but the extent of measurement variability and how volumetrics can be implemented in a clinical setting are unknown. Additionally, manual measurement techniques can be time-consuming and susceptible to inter-provider variability. Despite the absence of volumetry in the standard evaluation of PD, assessment of volume loss may be a valuable measurement for characterizing penile deformities and tracking efficacy of treatments. Being able to accurately, precisely, and efficiently include volumetry in workup may prove useful for improving care for PD.

One of the benefits of this study is the implementation of a 3D structured light scanner as a substitute for the traditional goniometer in characterization of PD deformities. Our results suggest 3D image capture is an effective tool to improve accuracy of measurements of the penis. Volume and angle of curvature showed statistically significant improvement compared to manual assessment. Previous studies have suggested the use of image capture in the evaluation of PD can improve accuracy and precision and limit subjectivity [7, 21]. Another benefit is that digital images can be magnified to a degree much superior to the naked eye, thereby allowing more precise and accurate determination of the point of maximal curvature and more subtle deformities. While some subjectivity exists with 3D imaging, these early results suggest that this method is worth exploring for the evaluation of penile deformities.

Several limitations were inherent to the design of this study. We used 3D modeling to create penile and cylindrical deformities with known measurements and therefore had “correct,” standardized values for comparison, but challenges when measuring a real penis could exist that are not present in a stationary, plastic model. This can include movement or loss of rigidity during image capture. Furthermore, this study evaluated only five 3D models, which necessarily excludes other presentations of penile deformities. More extreme models with either less or more severe bending, differences in length, and varying volumetric deformities could limit this study’s generalizability. Finally, although all participants received identical training prior to participating in the study, a separate analysis accounting for experience was not conducted.

## Conclusion

We conducted a proof of concept study in order to assess accuracy and variability in the measurement of length, angle, and volume of 3D cylindrical and penile models using 3D imaging technology. We found that length as a measurement for PD did not vary between providers in terms of precision and showed remarkable accuracy compared to 3D image capture using a structured light scanner, as well as to the standardized, predetermined length of all models. Manual measurements of penile volume and angulation showed significant variability and were limited in accuracy compared to computational measurements performed on 3D light scanned models. To our knowledge, this is the first study to assess accuracy and precision of traditional provider measurement methods of PD compared to digitally standardized, pre-determined values, and the first study to assess image capture accuracy of the Artec Space Spider structured light scanner compared to standardized values for PD. Our findings contribute to the existing literature that inter-provider variability may account for statistically significant pitfalls in accurate characterization of penile deformities in the clinic that can lead to adverse outcomes regarding patient satisfaction, condition severity, and treatment tracking. We have ongoing studies using this technology to look at pre and post-treatment evaluations of penile deformities to more objectively assess efficacy of different treatment modalities. The next step for this work is to translate this technology and workflow to human patients to improve the armamentarium of PD diagnostics.
